# Severe COVID-19 Infection in an Infant With 8p Inverted Duplication/Deletion Syndrome: Is Vaccination Still a Debate?

**DOI:** 10.7759/cureus.45060

**Published:** 2023-09-11

**Authors:** Andrew M Joseph, Monica Karas, Ernesto Joubran, Yaseen O Ramadan, Blakley A Fowler

**Affiliations:** 1 Osteopathic Medicine, Nova Southeastern University Dr. Kiran C. Patel College Of Osteopathic Medicine, Davie, USA; 2 Osteopathic Medicine, Nova Southeastern University Dr. Kiran C. Patel College of Osteopathic Medicine, Davie, USA; 3 Dr. Kiran C. Patel College of Osteopathic Medicine, Nova Southeastern University, Fort Lauderdale, USA; 4 Internal Medicine, HCA Florida Westside Hospital, Fort Lauderdale, USA; 5 Pediatric Medicine, Magnolia Regional Health Center, Corinth, USA

**Keywords:** covid-19, pediatric management, dexamethasone, remdesivir, vaccination

## Abstract

Despite having a milder course of coronavirus disease 2019 (COVID-19) in comparison to adults, children are at risk for more significant complications, including acute neurological, renal, respiratory, and cardiovascular complications. Acute complications can manifest as encephalopathy, renal injury, interstitial pneumonia, and heart failure. However, the most severe complication is multisystem inflammatory syndrome in children, which often requires intensive care to manage the subsequent respiratory failure. Moreover, children with comorbidities such as chronic lung disease, neurological disorders, and cardiovascular disease are at an elevated risk of morbidity and mortality. Here, we present the case of an 11-month-old white female patient, previously unvaccinated against COVID-19, with chronic lung disease and the 8p inverted duplication/deletion (Inv dup del (8p)) syndrome who suffered from a severe COVID-19 infection. Initially presenting to the pediatric clinic with nasal congestion and respiratory distress, the patient’s condition rapidly deteriorated which necessitated immediate transfer to the nearest pediatric tertiary center. There, she was mechanically ventilated, received dexamethasone and remdesivir, and was hospitalized for 26 days, nine of which were in the pediatric intensive care unit. To date, there is no current literature on Inv dup del (8p) syndrome as a predisposing factor for severe COVID-19 infection. Therefore, further investigation is needed to determine if Inv dup del (8p) can predispose a patient to having a severe COVID-19 course.

## Introduction

The coronavirus disease 2019 (COVID-19) pandemic profoundly impacted the medical community. It took many lives and devastated many others. An aspect of the pandemic not as often discussed is the impact of the disease on the pediatric population. As of July 2020, among all COVID-19 cases reported in the European Union/European Economic Area and the United Kingdom, only 4% were children or adolescents. Among them, 24% were under five years of age, 32% were between five and 11 years, and 44% were between 12 and 18 years [1]. A US study reported cumulative hospitalization rates of 19.1 per 100,000 infections among unvaccinated children and 9.2 per 100,000 infections among vaccinated children aged 5-11 years from December 2021 to February 2022 [2]. Among hospitalized children, 15% needed admission to the intensive care unit (ICU) [1]. However, many of the pediatric patients requiring hospitalization had one or more comorbidities, including chronic lung disease (18%), neurologic disorders (14%), and immunocompromised conditions (5.4%) [3-5]. By May 2022, COVID-19 was responsible for 1.7% of all deaths among children aged six months to four years, emerging as one of the leading causes of death in this age group during the pandemic [4].

Symptomatic pediatric COVID-19 patients present with a clinical profile similar to that of adults. The most frequently reported symptoms include fever (46%) and cough (37%) [3]. A unique concern in pediatric patients, however, is the most feared complication of COVID-19, the multisystem inflammatory syndrome in children (MIS-C) [3]. MIS-C is a severe delayed hyperinflammatory condition occurring in children and adolescents two to six weeks after a previous COVID-19 infection [6]. It is characterized by fever, elevated laboratory markers of systemic inflammation, and dysfunction in multiple organ systems, including cardiovascular, mucocutaneous, gastrointestinal, hematologic, neurologic, and renal involvement [6]. Some patients may also exhibit radiographic pulmonary abnormalities, possibly indicating associated pulmonary hyperinflammation, respiratory failure (both of which phenotypically overlap with COVID-19 viral pneumonia), or cardiogenic pulmonary edema [6,7]. Patients with MIS-C are often critically ill, with most requiring admission to an ICU, and 1-3% needing extracorporeal membrane oxygenation (ECMO) [7,8]. Mortality among MIS-C patients has been estimated at 1-2% [9].

We present the case of a patient with 8p inverted duplication/deletion (Inv dup del (8p)) syndrome, which is a rare structural rearrangement of the short arm of chromosome 8 [10]. To date, just over 100 cases of pediatric patients with Inv dup del (8p) syndrome have been published [10]. Clinical manifestations of Inv dup del (8p) syndrome vary significantly, but common symptoms encompass mild-to-severe intellectual disability, characteristic dysmorphic facial features, and central nervous system malformations such as hypoplasia or agenesis of the corpus callosum (67%) [10]. Additionally, this syndrome predisposes the child to other complications such as cardiovascular diseases (65%), musculoskeletal problems (59%), hypotonia (88%), and seizure disorder (55%) [10]. Inv dup del (8p) syndrome can also present with recurrent upper and lower respiratory tract infections, along with distinct dysmorphic facial features, including microcephaly, a large and prominent forehead, mildly arched eyebrows, deep-set eyes, hooded eyelids, full cheeks, a wide mouth, and micrognathia [10]. As such, the purpose of this case report is to discuss the management of severe COVID-19 in a pediatric patient with rare predisposing factors such as Inv dup del (8p) syndrome not reported in the literature.

## Case presentation

An 11-month-old white female infant with a past medical history of Inv dup del (8p) syndrome presented to a pediatric clinic, accompanied by her parents, due to concerns about respiratory distress for the past three days. Her past medical history consisted of agenesis of the corpus callosum, ventriculomegaly, atrial and ventricular septal defects, micrognathia, febrile seizures, and a history of respiratory syncytial virus (RSV) bronchiolitis. Her past surgical history included a tracheostomy tube placement at three days old, a tracheostomy tube change at one month old, and a gastrostomy tube placement at five weeks of age. Moreover, she depended on a gastrostomy tube and had a tracheostomy placed on day three of life, set to a baseline continuous rate of 3 L/minute oxygen with a 25% fraction of inspired oxygen (FiO_2_). According to her parents, she was exhibiting increased mucus secretions, nasal congestion, increased work of breathing, persistent cough, and audible wheezing. She was also experiencing intermittent oxygen desaturations of 50-60%, which required an increase from baseline to 20 L/minute of oxygen set to an FiO_2_ of 25%. Furthermore, the patient was up to date on all vaccinations but had not received the COVID-19 vaccine or boosters because the parents were afraid of the possible risks given the patient’s complicated medical history.

During the initial encounter, the patient was afebrile and tachypneic at a respiratory rate of 60 breaths per minute, which was increased from the normal reference range of 20-30 breaths per minute for her age group. On the physical examination, the patient appeared ill and was less playful and active compared to her baseline. She was in respiratory distress, tachypneic with subcostal retractions, and desaturating intermittently. The G-tube was noted with a clean site, and a tracheostomy collar was also noted at the anterior neck which was connected to an oxygen tank.

Due to the patient’s complex respiratory history and her current respiratory symptoms, an influenza A and B polymerase chain reaction (PCR) test, an RSV PCR test, and a COVID-19 nucleic acid amplification test were conducted in the office. The infant tested positive for COVID-19 and negative for RSV and influenza A and B.

While in the pediatric clinic, the patient rapidly progressed to respiratory failure due to increased work of breathing, ill appearance, and intermittent oxygen desaturations. Consequently, she was immediately transferred to the nearest tertiary pediatric center to receive escalated care for her respiratory failure, positive COVID-19 result, and complex past medical history.

**Figure 1:**
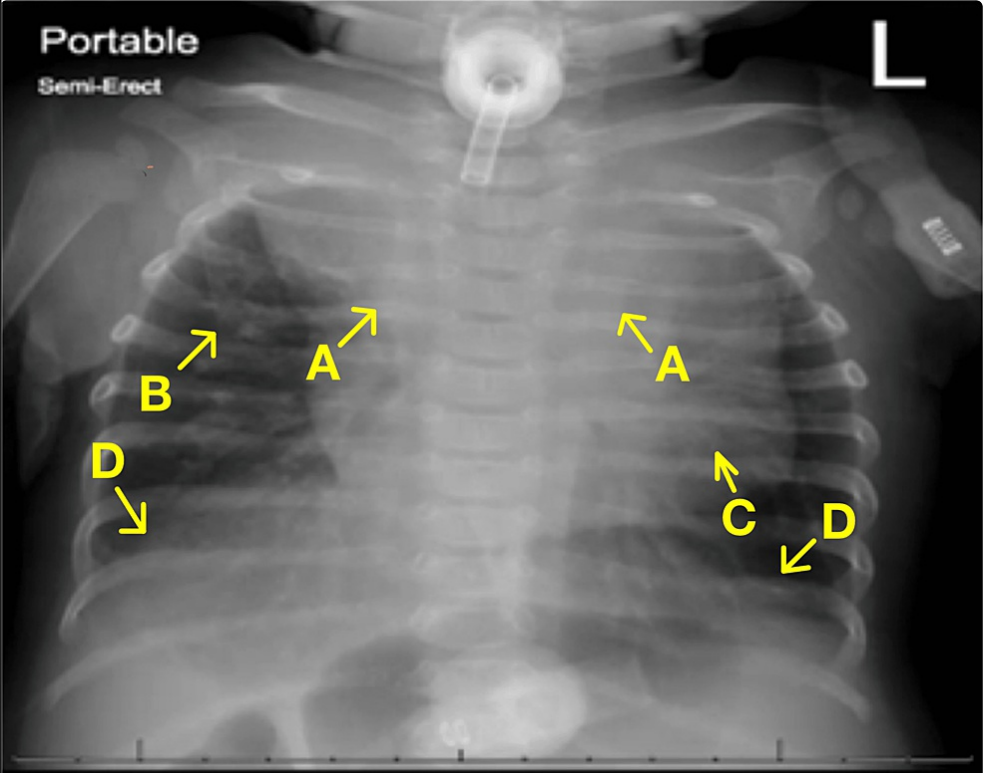
Chest X-ray of the infant on admission to the tertiary pediatric center. (A) Perihilar atelectasis. (B) Interstitial edema. (C) Cardiomegaly with enlarged cardio-thymic silhouette. (D) Bibasilar atelectasis.

Upon arrival at the tertiary pediatric center, an initial venous blood gas analysis revealed a pH of 7.37, a partial pressure of carbon dioxide of 62 mmHg, and a bicarbonate level of 36 mmol/L. These findings indicated respiratory acidosis with compensatory metabolic alkalosis. Additionally, a point-of-care glucose measurement showed a reading of 83 mg/dL. The infant’s chest X-ray, shown in Figure 1, revealed significant perihilar, retrocardiac, and bibasilar streaky/patchy areas of atelectasis, interstitial edema, and cardiomegaly with an enlarged cardio-thymic silhouette.

The patient’s initial laboratory results were significant for elevated neutrophils, lymphocytes, and monocytes. Table 1 presents all the patient’s initial laboratory values and compares them to their corresponding normal reference ranges.

**Table 1:** Initial complete blood count with differential on arrival to the tertiary pediatric center (bolded indicates abnormal value).

Blood component/Electrolyte	Patient’s value	Reference range
Hemoglobin	12.5 g/dL	10.0–15.0 g/dL
Hematocrit	40.3%	32.0%–43.0%
White blood cell count	10.0 × 10^3^/µL	5.0–15.0 × 10^3^/µL
Red blood cell count	4.6 × 10^6^/µL	4.0–5.5 × 10^6^/µL
Neutrophils	65.4%	15.0%–45.0%
Lymphocytes	18.1%	47.0%–77.0%
Monocytes	15.0%	0%–2.4%
Eosinophils	0.4%	0%–4%
Basophils	0.6%	0%–1.2%
Absolute granulocytes	4.16 × 10^3^/µL	1.8–7.1 × 10^3^/µL
Absolute immature granulocytes	0.05%	0%–0.5%
Ionized calcium	1.33 mmol/L	1.2–1.38 mmol/L

Given her presentation, the patient was placed on 15 L of continuous oxygen at 40% FiO_2_ through her tracheostomy tube, and her oxygen saturation briefly improved to above 90%. Despite administering albuterol and changing her tracheostomy tube, the patient did not improve. As such, she was placed on mechanical ventilation through her tracheostomy. Given her medical history, the potential for deterioration, and her COVID-19 infection, she was admitted to the pediatric intensive care unit (PICU) for further observation and management.

Pediatric intensive care unit course

Once in the PICU, the patient was initiated on ventilator support using the Synchronized Intermittent Mandatory Ventilation-Percutaneous Cardio-Pulmonary Support Trilogy system. Eventually, the patient’s condition stabilized with the following settings: a respiratory rate of 20 breaths per minute, positive inspiratory pressure of 18, positive end-expiratory pressure of 6, pressure support of +10, and FiO_2_ of 40%. These settings were selected to meet the patient’s increasing respiratory demands and address her hypoxemia. Once stabilized on the ventilator, the patient was administered a 10-day course of dexamethasone and remdesivir as part of her COVID-19 treatment. Overall, the patient remained in the PICU for nine days before being transferred to the intermediate care unit (IMCU).

Intermediate care unit course

The patient’s diagnosis upon arrival in the IMCU was an acute-on-chronic respiratory failure secondary to COVID-19 pneumonitis. Despite receiving full ventilator assist support, she continued to experience elevated carbon dioxide levels and oxygen desaturations to 50-60% due to being awake, having persistent coughing spells, and appearing uncomfortable. As a result, she was extubated and placed on spontaneous bilevel positive airway pressure (BiPAP) support, a form of noninvasive ventilation, that provided her considerable comfort. Her capillary blood gas (CBG) values improved, allowing her to eventually transition to continuous positive airway pressure (CPAP), a de-escalated form of noninvasive ventilation. The minimum CPAP settings that benefited the patient included +6 cmH_2_O and a rate of 1-3 L/minute of oxygen. This was made evident by the patient’s latest CBG values, which were within normal limits. Considering the normal CBG values and the patient’s comfort, she was prepared to be discharged home with a CPAP machine set to the same settings. After 17 days in the IMCU and a total of 26 days at the hospital, the patient was discharged home with home care provided by two trained caregivers.

## Discussion

This report presents the case of a patient with Inv dup del (8p) syndrome who suffered from a severe COVID-19 disease course leading to respiratory failure. The differential diagnoses included COVID-19 pneumonitis, asthma exacerbation with superimposed bacterial pneumonia, and viral bronchiolitis. She was then placed on mechanical ventilation and managed with dexamethasone and remdesivir. As she improved, she was transitioned from mechanical ventilation to BiPAP and eventually CPAP. She was then discharged home with her own CPAP and home care provided by two trained caregivers after a 26-day hospital stay. The patient’s Inv dup del (8p) syndrome or lack of COVID-19 vaccination might have increased her risk of a prolonged COVID-19 infection. Additionally, the patient’s past medical history of chronic lung disease and recurrent respiratory tract infections could have predisposed her to a severe COVID-19 course. However, it cannot be determined that these factors definitively led to her severe disease. As such, further investigation is needed to determine how, if at all, Inv dup del (8p) increases the risk of severe COVID-19.

Severe pediatric COVID-19 infection

Pediatric patients with severe COVID-19 infection should first be assessed for respiratory distress or failure [4,11]. The Pediatric Acute Lung Injury Consensus Conference criteria can be utilized to evaluate and guide the management of acute respiratory distress syndrome (ARDS) in pediatric COVID-19 patients [10]. Pediatric ARDS develops within seven days of infection and results in respiratory failure confirmed through chest imaging that reveals new infiltrates [11]. In special populations, such as our patient, with chronic lung disease, acute deterioration in oxygenation from the baseline leading to oxygen desaturation meets the criteria for pediatric ARDS [11]. Other signs and symptoms of pediatric ARDS exhibited by our patient included tachypnea, hypoxemia of less than 90%, and labored breathing [11].

Risk factors that can place pediatric patients under two years old at risk for a severe course of COVID-19 infection include chronic lung disease, neurologic disorders, cardiovascular disease, prematurity, and airway abnormality [12]. Our patient manifested several of these risk factors, due to Inv dup del (8p) genetic syndrome, including chronic lung disease, agenesis of the corpus callosum, prematurity, ventriculomegaly, atrial and ventricular septal defects, and micrognathia requiring a tracheostomy.

Pediatric COVID-19 management

For pediatric patients with COVID-19 infection and hypoxemia, supplemental oxygen through a nasal cannula can be utilized to achieve a target oxygen saturation of 92-96% [11]. If hypoxemia persists, patients can be escalated to a humidified high-flow nasal cannula (HFNC) to maintain an oxygen saturation to a fraction of inspired oxygen (SpO_2_/FiO_2_) ratio of greater than 264 [4,11]. In adults with COVID-19, awake self-prone positioning combined with HFNC has shown improvements in hypoxemia [11]. However, fewer studies have confirmed this noninvasive strategy in the pediatric population [11].

If hypoxemia continues despite HFNC and there is increased work of breathing, pediatric COVID-19 patients should be placed on noninvasive positive pressure ventilation (NIPPV) such as CPAP or BiPAP [4,11]. While recommendations do not place either HFNC or NIPPV as the first line of management, NIPPV is recommended as the standard of care, especially in pediatric COVID-19 patients with chronic lung disease such as status asthmaticus, as seen in our patient [4,11]. If NIPPV does not successfully increase oxygen saturation to above 92% and improve the work of breathing within 60 minutes, or sooner if the patient’s status deteriorates, the patient should be intubated [11].

Mechanical ventilation should aim to achieve a target expiratory tidal volume of 5-7 mL per kilogram ideal body weight, inspiratory plateau pressure below 28-32 cmH_2_O, driving pressure under 15 cmH_2_O, and permissive hypercapnia [11]. Positive end-expiratory pressure (PEEP) is recommended to be initiated at 5 cmH_2_O and titrated to the lowest possible PEEP/FiO_2_ ratio to maintain an SPO_2_ of 92 to 96% for moderate PARDS [11]. It is recommended to individualize the PEEP titration to each pediatric patient through stepwise trials of PEEP adjustments to optimize oxygenation [11]. Prone positioning is recommended for persistent hypoxemia despite titration of PEEP [11].

After securing the airway, medications are available for additional management of severe COVID-19 infections in pediatric patients. Remdesivir has received Food and Drug Administration approval for treatment in hospitalized COVID-19-infected adults and children [12]. This intravenous nucleotide prodrug inhibits viral replication by binding to viral RNA-dependent RNA polymerase, causing premature termination of RNA transcription [12]. To maximize therapeutic results, antiviral medications should be initiated early in the viral life cycle to maintain low overall viral levels [12]. The Infectious Diseases Society of America and the National Institutes of Health (NIH) recommend using remdesivir in hospitalized COVID-19 patients who require supplemental oxygen but are not undergoing mechanical ventilation [12].

Remdesivir should not be administered to newborns with serum creatinine levels under 1 mg/dL or to hospital patients who are at least 29 days old and have an estimated glomerular filtration rate below 30 mL/minute [12]. Remdesivir is typically well tolerated and has a manageable list of adverse effects [12]. The most commonly reported adverse effect of remdesivir is a rise in alanine aminotransferase (ALT) [12]. Usually, this ALT elevation is transient and returns to normal after therapy is stopped [12]. Hepatic function should, therefore, be evaluated as clinically necessary during treatment [12].

Dexamethasone has also been used in the management of severe COVID-19 infections in pediatric patients [11]. Specifically, dexamethasone is recommended in hospitalized pediatric patients requiring respiratory support such as HFNC, NIPPV, or invasive mechanical ventilation [13]. The ROCOVERY trial conducted among adults with COVID-19 requiring supplemental oxygen revealed that dexamethasone treatment improved survival and decreased the number of deaths to 23.3% compared to 26.2% for patients on standard of care [13]. Dexamethasone, a corticosteroid, targets the hyperinflammatory state induced by severe COVID-19 infection [13]. In pediatrics, the dose regimen is 0.15 mg/kg/dose with a maximum of 6 mg dose once daily for 10 days or until hospital discharge, whichever comes first [13].

In our patient, both dexamethasone and remdesivir were administered for the treatment of COVID-19. Observational studies have demonstrated that remdesivir administered with dexamethasone is more beneficial than dexamethasone alone [13]. The combination of the two has been shown to improve 30-day mortality to 1.3% compared to 16% on dexamethasone alone and increase viral clearance to a median of six days compared to 16 days [13]. The benefits may be attributed to the inhibition of viral replication in the setting of immunosuppression, thereby impacting both the virus and the hyperinflammatory state simultaneously [13].

Although not administered to this patient, immunomodulatory agents have also shown benefit when added with dexamethasone for pediatric patients requiring HFNC or NIPPV [13]. Examples of immunomodulatory agents that have been studied for COVID-19 infections include sarilumab, tocilizumab, and baricitinib [11]. Sarilumab, an interleukin 6 (IL-6) inhibitor, has been specifically not recommended for pediatric use in COVID-19 [13]. Tocilizumab, also an IL-6 inhibitor, has been shown to reduce hospital mortality in adult ICU patients with severe COVID-19 infection when added with dexamethasone in the REMAP-CAP and RECOVERY trials [13]. Baricitinib, a JAK1/JAK2 inhibitor, has also been shown to decrease mortality in adult COVID-19 patients in the COV-BARRIER trial [13]. As none of these immunomodulatory agents have been studied in pediatric patients, their use is limited to pediatric patients on mechanical ventilation or ECMO [13].

## Conclusions

This report presents the case of an unvaccinated 11-month-old white female with chronic lung disease and Inv dup del (8p) syndrome who developed a severe COVID-19 infection that was managed in the PICU by mechanical ventilation, dexamethasone, and remdesivir. Although the patient’s genetic condition and chronic lung disease may have played a role, it cannot be concluded that these factors definitively caused her severe COVID-19 course. It is also not reasonable to definitively assume that receiving the vaccine would have prevented the patient from requiring hospitalization. Because COVID-19 impacts a vast array of body systems, the complexity of individual cases must be further investigated from a vaccination standpoint. Further investigation is needed to determine the effects of COVID-19 vaccination in all children older than six months of age with factors that place them at an increased risk of a severe COVID-19 disease course.
